# How Much Sugar Do We Eat!

**Published:** 1854-12

**Authors:** 


How much Sugar do we Eat!—A western paper states, that 1853, there were consumed in this country about 705,000,000 pounds of cane sugar, and 27,000,000 pounds of maple sugar. This gives more than 24 pounds of cane sugar and one pound of maple sugar to every man, woman and child. This does not include molasses or honey. If this sugar were put into barrels holding 200 pounds, and each barrel occupied the space of three square feet only, it would require 336 acres of land for it to stand upon. The barrels, if placed in a row, would reach 220 miles. If the sugar was put up in paper packages of five pounds each, it would require 146,400,000 sheets of wrapping-paper; and if only a yard of string was used to each package, there would be required 439,200,000 feet, or 83,000 miles of string—more than three times enough to go round the world. If every retail clerk sold a hundred pounds of sugar each day, it would require nearly 25,000 clerks to sell it all in a year. If the dealers, wholesale and retail together, made a profit of only two cents a pound on this sugar, these profits would amount to nearly $15,000,000.

